# Co-existence of AMF with different putative MAT-alleles induces genes homologous to those involved in mating in other fungi: a reply to Malar et al.

**DOI:** 10.1038/s41396-021-00979-x

**Published:** 2021-05-03

**Authors:** Ivan D. Mateus, Soon-Jae Lee, Ian R. Sanders

**Affiliations:** Department of Ecology and Evolution, University of Lausanne, Lausanne, Switzerland

**Keywords:** Fungal ecology, Microbial ecology

The commentary by Malar et al. [1] raised four criticisms of our study [2]. (1) Observed upregulated genes were not the closest orthologs of genes involved in mating in other fungi. (2) Reanalysis of RNA-seq data failed to detect upregulation of 18 *R. irregularis* genes during co-existence of two *R. irregularis* isolates in cassava. (3) An adjusted probability of <0.1 led to a substantial number of false positives. (4) There was no evidence that two *R. irregularis* isolates showed divergence in a putative MAT-locus indicating mating compatibility. We consider that there are serious errors in the assumptions and rationale used for the re-analyses, a mis-interpretation of the original data, as well as the discarding of important data without any justification.

## Gene homology

Although Malar et al. “do not exclude the possibility that the genes identified by Mateus et al. are involved in mating,” they qualify the homology inference between genes differentially expressed in the co-inoculation treatment and genes involved in mating in other fungal species as “spurious evolutionary relationships” or “not the best ortholog”. Those statements imply that they attach no importance to the demonstrated sequence homology relationships identified in Mateus et al. Orthology does not necessarily imply conservation of gene function and genes with equivalent functions are not necessarily orthologs [3]. Therefore, it is misleading to assume that two genes have the same function when interpreting the role of a “best candidate ortholog” identified in silico. Moreover, relying only on an in silico search for exploring orthologs can lead to serious problems for inferring function as none of the search algorithms are free from bias if subfunctionalization or neofunctionalization events occurred among the homologs.

Malar et al. have not considered, or have misunderstood, the experimental evidence on gene expression in interpreting their homology search. It is not surprising that their “best homologs” were not upregulated, because we already saw that those genes were not upregulated in the original dataset. Our approach comprised performing an experiment to identify genes that were specifically upregulated when two isolates coexisted in planta. We then identified their putative function by homology. We did not look at whether the genes were the closest orthologs. However, we discussed the limitations of an homology approach to identify gene function [2]. To our surprise, a consistent set of 20 genes was upregulated in the co-inoculation treatment in different host plants, and 9 of these 20 (upregulated in more than one host plant) shared the common feature of homology to genes involved in different steps of mating in other fungal species (Figs. 3 and 4 of Mateus et al.).

Malar et al. claim the identification of hundreds of hits of the 18 genes differentially expressed in Mateus et al. “against the high-quality protein databases from the JGI Mycocosm Rhiir2” (referring to the protein database “Rhiir2” of *R. irregularis*). In fact, Malar et al. compared the 18 genes against “all protein gene catalogs of fungal species from the JGI fungal genomic resource” comprising 1318 taxa. The interpretation of the number of hits on a such large dataset is misleading because if a gene is highly conserved across the fungal kingdom, we would expect hundreds of hits in this database. In contrast, if an *R. irregularis* gene is highly specific to the Glomeromycotina taxa, we would expect very few hits (because there are less Glomeromycotina genomes in the database). Consequently, the number of hits in Table 1 from Malar et al. reflect the size of the database used and how conserved a given gene is, rather than whether a gene is from a large gene family. Malar et al. identified the so-called “closest ortholog” in *R. irregularis* of fungal mating genes from other fungal species by showing the “best hit” using OrthoMCL. However, differentiating paralogs from orthologs is a complicated task, in very distant species, especially if the organisms are highly paralogous. A more cautious analysis for each gene, including a confirmation that they are located in similar genomic locations, would lend more certitude that a given gene could be an ortholog. Consequently, the evaluation of RNA expression of their “best hit” remains incomplete in terms of the effort to find the best orthologs.

## Reanalysis of RNA-seq data

From the original data, Malar et al. [1] stated that some genes were upregulated in isolate DAOM197198 vs. the co-inoculation treatment and downregulated in isolate B1 vs. the co-inoculation treatment (a term they called “inconsistency”). This is entirely incorrect. The consistency of the response across replicates, treatment comparisons and in different host plants is evident from Figs. 1b–d, 2 and Supplementary Tables 6–9 of Mateus et al. [2]. Our only explanation is that Malar et al. [1] have simply misunderstood the values of fold-change in Supplementary Table 6 of Mateus et al. [2]. Positive fold-change values occurred in the comparison (B1 vs. co-inoculation) and negative values in the comparison (co-inoculation vs. DAOM197198). However, those values are negative simply because of the alphabetic order between the treatments and how the software processes them. For the same reason, the analyses on meiosis-specific genes by Malar et al. (Supplementary Table 3 [1]) actually demonstrate “consistency” but they claimed it as “inconsistent.” We can only conclude that they misread the output of DEseq2.

Malar et al. appear to have made another important error in their reanalysis. They report the comparisons of treatments DAOM197198 vs. co-inoculation and B1 vs. co-inoculation. In fact, Malar et al. have not reported the B1 vs. co-inoculation comparison at all. The supplemental tables, as well as the raw data deposited in GitHub (GitHub repository: madhubioinfo), show that they indeed performed a two-way comparison of DAOM197198 vs. co-inoculation with DEseq2 but then a comparison with the three treatments B1, co-inoculation, and DAOM197198 (raw data from GitHub repository: madhubioinfo). The consequence of the three treatments as input in the DEseq2 analysis is that the software compares the first and the third treatments. In their reanalysis, Malar et al. actually compared B1 vs. DAOM197198, which completely invalidates their claims.

Malar et al. claim to have found specific upregulation in only two putative orthologous genes in their reanalysis of the RNA-seq data. But they have discarded data without giving any scientific justification. In Supplementary Table 2, Malar et al. appear to have manually edited several cases with: “N.A = cannot be calculated based on mapped reads” even though the analysis should always yield a value, and did in our analysis. There is no explanation given about why they considered expression values could not be calculated, especially for only one comparison of a given gene (DAOM197198 vs. Co-inoculation) but not the other comparison of the same gene (B1 vs. co-inoculation). Consequently, the omission of those values that ensured their reanalysis would provide a different conclusion from that of Mateus et al.

## Adjusted probability threshold

As clearly stated in Mateus et al., we used an adjusted probability of <0.1 to maximize the identification of interesting genes. However, the change of probability threshold does not change the main finding that a large number of genes upregulated are homologs of genes involved in fungal mating. Eleven, of the 18 highlighted genes, displayed an adjusted probability <0.05 in both comparisons, and the other 7, an adjusted probability below 0.05 in one comparison and between 0.05 and 0.1 in the other comparison (Supplementary Table 6 [2]). Furthermore, we observed that several upregulated genes also showed an adjusted probability below 0.05 in more than one host plant (Fig. 3 and Supplementary Files 8 and 9, from Mateus et al.). This suggests that the experimental evidence of upregulated genes in the co-inoculation treatments is robust.

## Mating compatibility of the fungal isolates

The authors questioned the possibility of mating compatibility between isolates B1 and DAOM197198, because no sequence information at the putative MAT-locus [4] was provided. Despite the lack of any published experimental evidence that AMF are compatible only when carrying different alleles at this locus, we tested if the two isolates carried different alleles. Indeed, we confirm that isolate B1 displays a MAT-6 putative MAT-locus (see LC602501 for NCBI sequence accession) and that of isolate DAOM clusters with MAT-4 type [4]. Consequently, isolates B1 and DAOM197198 harbor different alleles of the “putative” MAT-locus.

To conclude, Malar et al. asked “Where is the evidence?” To respond to their question, the evidence of specific gene upregulation during AM fungal co-existence is where Malar et al. chose not to see it.
